# Translation, validity and reliability of the Turkish Chronic Illness Job Strain Scale (CIJSS) in people with inflammatory arthritis

**DOI:** 10.1093/rap/rkaf142

**Published:** 2025-12-02

**Authors:** Gonca Bumin, Ezginur Gündoğmuş, Alan Tennant, Sevilay Karahan, Umut Kalyoncu, Yeliz Prior

**Affiliations:** Department of Occupational Therapy, Faculty of Health Sciences, Hacettepe University, Ankara, Turkey; Department of Occupational Therapy, Faculty of Health Sciences, Hacettepe University, Ankara, Turkey; Leeds Institute of Rheumatic and Musculoskeletal Medicine, University of Leeds, Leeds, UK; Department of Biostatistics, Faculty of Medicine, Hacettepe University, Ankara, Turkey; Department of Rheumatology, Faculty of Medicine, Hacettepe University, Ankara, Turkey; Centre for Human Movement and Rehabilitation, School of Health and Society, University of Salford, Salford, UK; Versus Arthritis/MRC Centre for Musculoskeletal Health and Work, School of Health and Society, University of Salford, Salford, UK

**Keywords:** job strain, reliability and validity, rheumatologic diseases, vocational rehabilitation

## Abstract

**Objective:**

This study aimed to adapt the Chronic Illness Job Strain Scale (CIJSS) into Turkish and evaluate its validity and reliability in individuals with rheumatologic conditions.

**Methods:**

The CIJSS was culturally adapted following Beaton’s protocol. Construct validity was assessed using Rasch analysis. Concurrent validity was examined through correlations with work-related [Work Limitation Questionnaire-Short Form (WLQ-SF), Rheumatoid Arthritis Work Instability Scale (RA-WIS), Work Productivity and Activity Impairment General Health V2.0 (WPAI-GH)] and health-related [Health Assessment Questionnaire (HAQ), Rheumatoid Arthritis Impact of Disease (RAID)] measures. Test–retest reliability was assessed 2 weeks later.

**Results:**

The CIJSS demonstrated high internal consistency (Cronbach’s α = 0.96) and excellent test–retest reliability (Spearman’s *r* = 0.886; ICC(2,1) = 0.88). The scale met Rasch model requirements for fit. Significant correlations were observed between the CIJSS total score and the WLQ total score (*r* = –0.61), WPAI scores (*r* = 0.19–0.47), HAQ (*r* = 0.33), and RAID (*r* = 0.61), supporting concurrent validity.

**Conclusions:**

The Turkish CIJSS is a valid, reliable, and culturally appropriate tool for assessing job strain in individuals with inflammatory arthritis. It can support both clinical assessment and vocational rehabilitation planning for Turkish-speaking populations.

Key messages

- The Turkish version of the CIJSS is a valid and reliable measure of job strain in individuals with inflammatory arthritis.
- Its strong correlations with established work and health measures demonstrate that the CIJSS captures how chronic illness affects participation in work.
- The CIJSS can support clinical assessments and vocational rehabilitation by identifying work-related challenges in Turkish-speaking people with rheumatologic conditions.

## Introduction

The World Health Organization (WHO) defines chronic disease as a long-term condition that typically progresses slowly over time [1]. Inflammatory arthritis (IA) is a chronic inflammatory condition that includes, but is not limited to, RA, axial spondyloarthritis (axSpA), and psoriatic arthritis (PsA). It is characterized by joint swelling, stiffness, fatigue, pain, psychological distress, and reduced mobility [2, 3]. Despite advances in pharmacological and surgical treatments, many individuals with IA continue to experience significant physical disability and psychosocial challenges [4]. Many face difficulties in maintaining employment and balancing work with daily energy demands [5]. Additionally, people with IA are at risk of job loss and long-term exclusion from the workforce, with up to 40% leaving work within the first few years of diagnosis [2]. Participation in paid work is important for identity, social inclusion, and a sense of purpose, but various aspects of work and the working environment can create challenges [6]. These include the nature of job tasks, relationships with colleagues, and work schedules. Job strain refers to an individual’s perception of the work environment as stressful. Both job-related stressors and job strain are recognized as key factors that influence health and the ability to remain in employment [7].

Job strain refers to physical and mental tension that arises when there is a mismatch between demanding job requirements and an individual’s ability to adapt and cope effectively, where psychological demands are high, and individuals have limited control or decision-making power in their work [8]. Managing chronic pain, fatigue, and physical limitations in the workplace is often challenging and can be perceived as highly stressful for individuals with IA who may experience different forms of job strain [9]. The unpredictable nature of symptoms adds further difficulty, with uncertainty being widely recognized as a significant stressor [10]. Uncertainty about how to manage the condition at work can create emotional tension, both in meeting current job responsibilities and in concerns about long-term job security [11]. Stress may also arise because arthritis symptoms are often invisible to others, who may be unaware of the person’s condition or may not understand the variability of their experience from day to day [9, 12].

Many tools have been developed to assess job stress, but none have been designed specifically to measure job strain in individuals with IA. Existing scales include the Work Stress Scale 20, which categorizes stressors into emotional or psychological factors, interpersonal relationships or daily challenges, physical demands, environmental influences, and life changes or social life events [13]. The General Work Stress Scale has been validated in nursing populations [14], and the Swedish Workload, Work Control, and Social Support Questionnaire, which covers workload, autonomy at work, and social support, was validated in male workers [15]. However, no Turkish-language scale currently assesses job strain specifically in people with chronic IA. The Chronic Illness Job Strain Scale (CIJSS), developed in Canada, was designed to address this gap. It measures job strain in relation to several aspects of working with arthritis, including symptom burden at work, job demands, work pace and scheduling, use of training, uncertainty about symptoms, interpersonal tension, access to workplace support, financial strain, and managing multiple roles [9]. The CIJSS was later adapted and validated in British English for use in employed individuals with RA, axSpA, OA, or fibromyalgia, and demonstrated strong reliability, with Cronbach’s alpha values ranging from 0.93 to 0.96 [16].

The aim of this study was to adapt the CIJSS for use in Turkish and to evaluate its reliability and validity in individuals with IA, specifically in RA, axSpA and PsA. In addition, the study aimed to assess internal consistency and Rasch analysis to examine other key psychometric properties of the scale.

## Methods

### Study design

The scale was culturally adapted into Turkish following the translation and adaptation guidelines outlined by Beaton *et al.* [17] and the process adhered to the Consensus-based Standards for the Selection of Health Measurement Instruments (COSMIN) checklist [18] (Supplementary File S1, available at *Rheumatology Advances in Practice* Online).

#### Phase 1: Translation, linguistic validation, and cross-cultural adaptation 

The CIJSS was translated from English into Turkish using a standard forward–backward translation process. Two independent native Turkish translators conducted forward translations. These were compared with identify discrepancies, including potential ambiguities in the original text or inconsistencies between versions. The translators worked together to synthesize a single reconciled version (T-12), informed by both translations and the original English questionnaire.

The T-12 version was then back-translated into English by two native English speakers fluent in Turkish, who were blind to the original version. The back-translated texts were examined for conceptual equivalence and consistency with the original scale. Following review of all versions, a final pre-test version of the Turkish CIJSS was developed, ensuring alignment with the source instrument and cultural appropriateness.

#### Phase 2: Implementation of psychometric tests

To evaluate the concurrent validity of the CIJSS by comparing it with measures of similar and related constructs, participants completed the Turkish version of the CIJSS along with a series of work and health-related questionnaires, described below.

### Participants

The study included 230 participants in total, comprising 30 individuals in phase 1 and a separate group of 200 individuals in phase 2. All were adults aged 18 years or older with a confirmed diagnosis of IA limited to RA, axSpA or PsA and in full-time or part-time paid employment. All diagnoses were confirmed by a rheumatologist. Individuals on medical leave due to long-term illness were excluded. Ethical approval was obtained from the Hacettepe University Health Sciences Research Ethics Committee (decision number: 2024/01-03). All participants provided written informed consent. To assess construct validity, a minimum of 150 cases per group was recommended [18]. To maximize the diversity of responses and allow for a dropout, we aimed to recruit 200 participants. For test–retest reliability, at least 79 repeat responses were required to detect a correlation of 0.7 as significantly different from a background correlation of 0.45, with 90% power at the 1% significance level [19].

Participants provided information on age, height, weight, body mass index, gender, diagnosis, duration of diagnosis, and duration of symptoms. Employment details included current working status (part-time or full-time), average weekly working hours, size of the workplace, and physical demands of the job. Additional data were collected on education level, marital status (married, divorced, living alone, or living with a spouse, partner, or family), and current medications.

#### Chronic Illness Job Strain Scale

The CIJSS was developed by Gignac *et al.* [9] to assess job strain in individuals with arthritis. It covers several key domains, including arthritis symptoms in the workplace, job demands and work scheduling, use of training, uncertainty related to symptoms, interpersonal tension, coping resources, job retention, financial concerns, and balancing multiple roles. The scale consists of 15 items rated on a 5-point Likert scale, where 1 indicates ‘not at all stressful’, and 5 indicates ‘extremely stressful’. Total scores range from 15 to 75, with higher scores reflecting greater perceived job strain. The scale has demonstrated high internal consistency, with a reported Cronbach’s alpha of 0.95 [16].

#### Work Limitation Questionnaire-Short form

The Work Limitation Questionnaire–Short form (WLQ-SF) measures the extent to which physical or emotional health problems have affected an individual’s ability to meet job demands over the past 2 weeks [20]. It consists of two items for each of four domains: time management, physical demands, mental-interpersonal demands, and output demands. Respondents rate each item using a 6-point Likert scale, where 1 represents ‘all of the time’ (100% of the time) and 6 indicates ‘does not apply to my job’. Completion takes approximately 10–15 min. The Turkish version of the WLQ-SF has demonstrated acceptable reliability, with a reported Cronbach’s alpha of 0.83 [21].

#### The Rheumatoid Arthritis Work Instability Scale

The Rheumatoid Arthritis Work Instability Scale (RA-WIS) is a 23-item questionnaire designed to assess work instability in individuals with RA by identifying mismatches between job demands and an individual’s functional capacity. Each item is answered as true or false, focusing on the perceived impact of RA on work functioning [22].

#### Work Productivity and Activity Impairment General Health V2.0

The Work Productivity and Activity Impairment General Health V2.0 (WPAI: GH) consists of six items that evaluate the impact of health problems on work productivity and regular daily activities. It includes questions related to sick leave, working hours, and perceived difficulties at work. The instrument produces four outcome measures, all expressed as percentages: time missed from work due to health problems, impairment while working, overall work productivity loss, and activity impairment outside of work. Higher scores indicate greater impairment and productivity loss [23].

#### Health Assessment Questionnaire

The Health Assessment Questionnaire (HAQ) is a 20-item instrument designed to assess the physical functioning of individuals with RA in daily activities [24]. It evaluates difficulties in performing routine tasks such as dressing, walking, and eating. The Turkish version of the HAQ has been validated and demonstrates excellent reliability, with a reported Cronbach’s alpha of 0.97 [25].

#### Rheumatoid Arthritis Impact of Disease scale

The Rheumatoid Arthritis Impact of Disease (RAID) scale measures the overall impact of RA on an individual’s daily life [26]. It consists of seven numeric rating scales, each ranging from 0 to 10, assessing domains such as pain, fatigue, and physical function. The total score is calculated by summing the individual domain scores, with higher values indicating greater disease impact. The Turkish version of the RAID has shown strong reliability, with a reported Cronbach’s alpha of 0.93 in individuals with RA [27].

Additionally, participants’ health-related conditions were assessed using a Numeric Rating Scale. This included self-reported ratings of confidence in working, mood, and severity of hand pain on a scale from 1 to 10, and overall health status on a scale from 1 to 5.

### Statistical analysis

All statistical analyses were conducted using IBM SPSS Statistics for Windows, Version 23.0 (IBM Corp., Armonk, NY, USA). Numerical variables were reported as mean ± S.D. and median (25th–75th percentile), while categorical variables were summarized as frequencies and percentages. The normality of numerical variables was assessed using graphical analysis and the Kolmogorov–Smirnov test. Due to non-normal distributions in WPAI: GH subscales, non-parametric tests (Spearman’s *r*) were applied in correlation analyses. In this study, correlation coefficient values were calculated according to Chan’s classification system. According to this, a coefficient of at least 0.8 indicates a very strong linear relationship, while values between 0.6 and 0.8 indicate a moderately strong relationship. When the coefficient is between 0.3 and 0.5, the relationship can be considered fair. Finally, coefficients less than 0.3 indicate a poor linear relationship [28].

The internal construct validity of the scale was examined through Rasch analysis [29]. This included test of invariance (differential item functioning [DIF]) across a range of contextual factors, including age, gender, diagnosis, medication use, body mass index, disease duration, and time (retest). Additional details related to the Rasch analysis are given in Supplementary File S2, available at *Rheumatology Advances in Practice* Online.

#### Concurrent validity

The relationships between the work-related comparator measures were examined to establish concurrent validity. Spearman correlation assessed the associations between the work-related questionnaires (WLQ-SF, RA-WIS, WPAI-GH) and the health-related questionnaires (HAQ, RAID).

#### Reliability

Test–retest reliability of CIJSS was assessed using the intraclass correlation coefficient (ICC) and the Wilcoxon test. Internal consistency was evaluated by calculating Cronbach’s α, with values of ICC ≥ 0.75 and Cronbach’s α  ≥  0.70 considered acceptable [30]. Corrected item–total correlations and item-deleted Cronbach’s α values were also examined for each subscale. Corrected item–total correlations above 0.30 were considered acceptable [19].

#### Responsiveness

The scale’s sensitivity to change was assessed by calculating the S.E.M. and the smallest detectable difference (SDD) at 95% CI (SDD). To assess the measurement precision, the S.E.M. was calculated using the formula S.E.M. = S.D. (pooled) × √ (1 − ICC). Additionally, the SDD was calculated using the formula SDD = 1.96 × √2 × S.E.M., which provides an estimate of the smallest change that can be considered statistically significant.

#### Floor and ceiling effects

The floor and ceiling effects of the scale were analysed. A floor and ceiling effect below 15% was considered acceptable, indicating that the scale effectively measures across a broad range of individuals without excessive clustering at extreme values [31].

## Results

Participants took part in cognitive debriefing interviews (*n* = 30) referred to as phase 1. These participants found all items of the CIJSS to be highly relevant. Following review by an expert panel, the final version of the scale was approved without requiring any wording changes. In phase 2, 200 participants were included in the study, excluding those who participated in phase 1, with an average age of 37.9 (S.D. = 10.6) and 66.5% being female (Table 1). In this sample, in line with the Assessment of SpondyloArthritis International Society (ASAS) framework, axSpA included both non-radiographic axial SpA (nr-axSpA) and radiographic axial SpA (r-axSpA, equivalent to ankylosing spondylitis), and these were grouped together within the ‘axial SpA’ category. One participant was categorized as the ‘undifferentiated spondyloarthritis’ (u-SpA). Medication use was categorized into conventional synthetic DMARDs (csDMARDs), biologic DMARDs (bDMARDs), and targeted synthetic DMARDs (tsDMARDs)(this latter category is not represented in Table 1). No significant differences were observed between drug types in terms of age (*F* = 0.12; df = 3.196; *P* = 0.948) or gender (χ^2^ = 2.458; df = 3; *P* = 0.483).

**Table 1:** Demographic information of phase 1 and phase 2 participants.

		Phase 1 (*n* = 30)		Phase 2 (*n* = 200)	
		Mean ± S.D.	Min–Max	Mean ± S.D.	Min–Max
Age (years)		43.0 ± 12.3	23–64	37.9 ± 10.6	19–66
Height (cm)		160.7 ± 31.2	152–188	168.6 ± 8.7	145–190
Weight (kg)		72.6 ± 13.0	53–105	70.3 ± 12.0	48–105
BMI		26.4 ± 4.5	20.9–39.1	24.7 ± 3.7	18.0–39.1
Duration of diagnosis		11.4 ± 10.7	1–35	9.2 ± 8.0	1–35
Duration of complaints		12.7 ± 10.6	1–36	10.6 ± 8.3	1–36
Average working hours per week		39.1 ± 11.7	8–60	42.3 ± 9.3	10–72
Confidence in working		6.56 ± 3.4	0–10	7.3 ± 2.9	0–10
Mood		4.7 ± 2.7	0–10	4.6 ± 2.7	0–10
Overall health status		3.0 ± 0.8	1–5	2.9 ± 0.8	1–5
Severity of hand pain		3.6 ± 3.1	0–10	4.3 ± 3.2	0–10
		**Frequency**	**Percent**	**Frequency**	**Percent**
Gender	Female	22	73.3	133	66.5
	Male	8	26.7	67	33.5
Diagnosis	RA	21	70	130	65
	axSpA	9	30	49	24.5
	u-SpA			1	0.5
	PsA			20	10.0
Workplace size	0–50 people	15	50	75	37.5
	50–100 people	3	10	40	20.0
	100+	12	40	85	42.5
Employment status	Full time	27	90	187	93.5
	Part-time	3	10	13	6.5
Physical demands of the job	Low	10	33.3	45	22.5
	Moderate	15	50	96	48.0
	High	5	16.7	59	29.5
Education level	Primary school	1	3.3	7	3.5
	Secondary school	2	6.7	7	3.5
	High school	5	16.7	23	11.5
	University	19	63.3	129	64.5
	Postgraduate	3	10	34	17.0
Marriage status	Married	19	63.3	134	67.0
	Divorced	2	6.7	20	10.0
	Living with parents	4	13.3	19	9.5
	Living alone	5	16.7	27	13.5
Medication type	None			6	3.0
	NSAID	1	3.3	6	3.0
	Steroid			9	4.5
	csDMARD	14	46.66	75	37.5
	tsDMARD	1	3.33	8	4
	bDMARD	9	30	52	26
	Neuropathic analgesic			1	0.5
	Biologic/biosimilar	1	3.3	15	7.5
	Steroids, NSAIDs			1	0.5
	NSAIDs, analgesics	4	13.3	27	13.5

The category ‘u-axSpA’ refers to undifferentiated spondyloarthritis, describing participants with features of spondyloarthritis who did not meet classification criteria for psoriatic or axial SpA. The axial SpA category includes both non-radiographic axial SpA (nr-axSpA) and radiographic axial SpA (r-axSpA, equivalent to ankylosing spondylitis), according to the Assessment of SpondyloArthritis International Society (ASAS) criteria.

Additional demographic characteristics are presented in Table 1.

The work and health assessment test results for phase 2 are presented in Table 2 (*n* = 200).

**Table 2:** Descriptive statistics for the phase 2 participants’ work and health measures scores (*n* = 200).

	Mean ± S.D.	Median [25th–75th percentile]
CIJSS total	43.93 ± 13.99	44 [34–54]
WLQ-TM	3.16 ± 1.16	3 [2.5–4]
WLQ-PD	2.45 ± 0.89	2.5 [2 3]
WLQ-MID	3.77 ± 0.87	4 [3–4.5]
WLQ-OD	3.52 ± 1.12	3.5 [3–4.5]
WLQ total	12.90 ± 2.39	13 [11.5–14.5]
WLQ percentage loss	15.04 ± 4.44	14.58 [11.85–18.58]
RA-WIS	12.04 ± 6.40	13 [7–17]
WPAI: GH1	13.14 ± 22.53	0 [0–18]
WPAI: GH2	44.00 ± 25.12	50 [20–60]
WPAI: GH3	34.92 ± 21.65	30 [20–50]
WPAI: GH4	48.80 ± 26.11	50 [30–70]
HAQ	13.27 ± 4.05	13 [10–17]
RAID	4.95 ± 2.23	4.8 [2.94–6.96]

CIJSS: Chronic Illness Job Strain Scale; WLQ: Work Limitation Questionnaire; TM: time management; PD: physical demands; MID: mental-interpersonal demands; OD: output demands; RA-WIS: Rheumatoid Arthritis Work Instability Scale; WPAI: Work Productivity Activity Impairment; HAQ: Health Assessment Questionnaire; RAID: Rheumatoid Arthritis Impact of Disease.

### Construct validity

In applying the data to the Rasch model, all item thresholds were ordered, indicating consistent progression across response categories (Supplementary File S4, available at *Rheumatology Advances in Practice* Online). The scale showed excellent targeting, with a mean person estimate of –0.011, which is close to the ideal mean of zero, indicating strong alignment between item difficulty and respondent ability (Supplementary File S5, available at *Rheumatology Advances in Practice* Online). The easiest threshold transition occurred between ‘not at all stressful’ and ‘a little stressful’ for the item, ‘Do your symptoms of your condition make your work…?’ The most difficult transition occurred between ‘quite a bit stressful’ and ‘extremely stressful’ for the item, ‘Is your current relationship with your co-workers affected because of your condition?’

Analysis of the baseline data at the item level showed only weak fit. There was no DIF across the contextual factors, and excellent reliability, but the scale was multidimensional. After grouping items, the CIJSS showed adequate fit to the model under a two super-item bi-factor equivalent solution which only required 7% of the variance to be discarded. This did show a reduction in reliability due to absorbing local item dependency in the data (i.e. correlated item residuals) [32]. Test–retest correlation on the metric at 0.89 was excellent, and the scale was shown to be invariant by country (Table 3). Full details of the Rasch analysis results are in Supplementary File S1, available at *Rheumatology Advances in Practice* Online.

**Table 3:** Fit of CIJSS to the Rasch model.

Analysis type	Residuals		Test of fit			Reliability		Dimensionality		DIF	Variance
	Item	Person	ꭕ	df	*P*	α	PSI	*t*-test %	LCI %		
Turkish											
1. Item-based	1.874	1.931	76.4	45	0.002	0.96	0.95	15.0	12.0	–	–
2. Super item	1.297	1.105	12.1	15	0.669	0.91	0.92	7.0	4.0	–	0.95
3. Test–retest super item	1.500	1.141	16.8	15	0.329	0.91	0.92	7.9	5.3	–	0.95
4. Test–retest two super items	2.154	0.994	3.5	6	0.747	0.85	0.90	3.2	0.7	–	0.93
Turkish–English											
5. English–Turkish	2.207	0.858	10.7	14	0.708	0.87	0.89	2.8	0.9	–	0.91
Ideal value	**<1.4**	**<1.4**			**>0.01**	**>0.7**	**>0.7**	**<5.0**	**<5.0**		**>0.9**

α: Cronbach’s alpha; DIF: differential item functioning; LCI: lower confidence interval; PSI: Person Separation Index.

Item–total correlations range from 0.654 to 0.827. The internal consistency coefficient of the scale is 0.96. When the Cronbach alpha values are examined when the item is deleted, it is seen that this value is similar for all items (Table 4).

**Table 4:** Item statistics of CIJSS.

	Mean	S.D.	Corrected item–total correlation	Cronbach’s alpha if item-deleted
CIJSS1	3.14	1.00	0.739	0.957
CIJSS2	2.98	1.10	0.820	0.956
CIJSS3	3.08	1.09	0.795	0.956
CIJSS4	3.07	1.10	0.757	0.957
CIJSS5	2.89	1.31	0.654	0.959
CIJSS6	2.93	1.30	0.767	0.957
CIJSS7	2.83	1.16	0.800	0.956
CIJSS8	2.75	1.24	0.797	0.956
CIJSS9	2.87	1.12	0.814	0.956
CIJSS10	2.80	1.27	0.776	0.957
CIJSS11	2.69	1.19	0.796	0.956
CIJSS12	2.37	1.12	0.762	0.957
CIJSS13	3.03	1.25	0.827	0.956
CIJSS14	3.23	1.11	0.745	0.957
CIJSS15	3.31	1.10	0.683	0.958

CIJSS: Chronic Illness Job Strain Scale.

### Concurrent validity

According to the Spearman correlation analysis, work limitations showed moderately strong negative correlations with WLQ time management (ρ = –0.617), output demands (ρ = –0.591), total score (ρ = –0.613), and percentage loss (ρ = –0.625). The correlation with WLQ mental demands was also moderately strong and negative (ρ = –0.528), while physical demands showed a fair positive correlation (ρ = 0.400). The RA-WIS demonstrated a poor correlation (ρ = –0.127).

Regarding WPAI outcomes, absenteeism (Score 1, *P* = 0.199) showed a poor positive correlation (ρ = 0.199), while presenteeism (Score 2, ρ = 0.452) and activity impairment (Score 4, ρ = 0.473) were in the fair positive range. Overall work productivity loss (Score 3, *P* = 0.262) also indicated a poor positive correlation (ρ = 0.262).

Among clinical measures, HAQ showed a fair positive correlation (ρ = 0.333), and RAID demonstrated a moderately strong positive correlation (ρ = 0.617). For patient-reported outcomes, confidence in working was fair negative (ρ = –0.352), while mood (ρ = 0.228) and overall health status (ρ = 0.221) showed poor positive correlations. Finally, the severity of hand pain was in the poor positive range (ρ = 0.294) (Table 5).

**Table 5:** Concurrent validity of the CIJSS with work and health measures.

	Spearman corr. coef.
WLQTMTOT	−0.617**
WLQPDTOT	0.400**
WLQMIDTOT	−0.528**
WLQODTOT	−0.591**
WLQTOTAL	−0.613**
WLQ percentage loss	−0.625**
RA-WIS	−0.127
WPAISCORE1	0.199*
WPAISCORE2	0.452**
WPAISCORE3	0.262**
WPAISCORE4	0.473**
HAQ	0.333**
RAID	0.617**
Confidence in working	−0.352**
Mood	0.228*
Overall health status	0.221*
Severity of hand pain	0.294**

WLQ: Work Limitation Questionnaire; TM: time management; PD: physical demands; MID: mental-interpersonal demands; OD: output demands; RA-WIS: Rheumatoid Arthritis Work Instability Scale; WPAI: Work Productivity Activity Impairment; HAQ: Health Assessment Questionnaire; RAID: Rheumatoid Arthritis Impact of Disease; corr. coef.: correlation coefficient.

* *P* < 0.01,

** *P* < 0.001.

### Test–retest reliability

There is no statistically significant difference between the first and second administration of the scale. The test–retest relationship between the two applications was high. SDD was obtained as 5.30 (Table 6).

**Table 6:** Test–retest reliability of CIJSS.

	T1	T2	*P* (Wilcoxon test)	Spearman correlation	ICC(2,1) (95% CI)	S.E.M.	SDD
CIJSS total	46 [34–58]	45 [35–60]	0.396	0.886**	0.885 (0.826–0.925)	3.65	5.30

ICC: intraclass correlation coefficient; SDD: smallest detectable difference.

** *P* < 0.001.

### Floor and ceiling effect

The scale has no floor or ceiling effect. (Floor effect: 1%; Ceiling effect: 0.5%.)

## Discussion

This study evaluated the validity and reliability of the Turkish version of the CIJSS in people with IA. The findings indicate high internal consistency and strong reliability, supporting its use in clinical and research settings.

Participants found the items clear, relevant, and culturally appropriate, requiring no modifications. This suggests successful adaptation for the Turkish population and supports content validity. Similar studies emphasize that ensuring linguistic and cultural alignment is essential for valid measurement [17].

Rasch analyses provided strong evidence of construct validity. Items demonstrated expected threshold ordering with only a 7% loss of variance, due to accommodating local item dependency [33]. The absence of DIF by age, gender, diagnosis, or medication use further supports the objectivity of the CIJSS.

Internal consistency was excellent (Cronbach’s α = 0.96), and test–retest reliability was high (Spearman’s *r* = 0.886; ICC = 0.88), exceeding recommended thresholds for clinical tools [31]. These results align with those of the British English CIJSS validation, where Cronbach’s alpha ranged from 0.93 to 0.96 and ICC from 0.92 to 0.96 across RA, axSpA, OA, and fibromyalgia [16].

Concurrent validity was confirmed through correlations with established work and health-related measures (WLQ, WPAI, HAQ, RAID). Negative correlations with WLQ subscales suggest that higher job strain is linked with greater work limitations [32, 34, 35], consistent with reports identifying time pressure and work demands as key contributors to strain in rheumatologic conditions [9, 36]. Positive correlations with RAID indicate that pain and fatigue are associated with increased job strain, directly affecting work performance [37, 38]. Moderate correlations with WPAI confirm that job strain contributes to reduced productivity, echoing prior findings [12].

Although correlations with WPAI (*r* = 0.19–0.47) and HAQ (*r* ≈ 0.33) were modest, this indicates that CIJSS captures related but distinct constructs rather than duplicating productivity or disability measures. The strongest associations were with WPAI2 and WPAI4 (presenteeism and activity impairment), suggesting that job strain relates more to difficulties performing work than to absenteeism. This aligns with evidence that presenteeism and activity impairment are major drivers of work disruption in rheumatologic disease [39, 40]. The weaker association with WPAI1 (absenteeism) suggests that factors such as workplace policies and coping styles may influence absence independently of perceived strain.

The fair correlation with HAQ implies that physical limitations such as pain, stiffness, and fatigue contribute to job strain, but CIJSS also reflects psychosocial factors (e.g. time pressure, mental demands, and job insecurity) absent from physical function measures [10, 39, 41, 42]. This distinction is clinically meaningful as it demonstrates that CIJSS provides complementary insights into the broader impact of chronic illness on work. Assessing work stress separately helps clinicians and occupational therapists identify individuals whose work participation is disproportionately affected and supports tailored interventions such as workplace adjustments, fatigue management, and coping strategies.

CIJSS scores also correlated with factors such as job security, mood, and hand pain severity, supporting that the scale captures a multidimensional construct influenced by physical, psychological, and social factors [43]. The very low floor (1%) and ceiling (0.5%) effects indicate good sensitivity and the ability to distinguish a wide range of job strain levels. Regular assessment of job strain can inform personalized return-to-work plans and targeted interventions [44]. The CIJSS therefore offers a comprehensive approach to understanding the work experiences of individuals with IA.

This study has several strengths. Construct validity was rigorously assessed using Rasch analysis, confirming the internal structure of the Turkish CIJSS and its fit with modern measurement standards. Cross-cultural measurement invariance with the British English version further supports comparability across populations.

However, some limitations should be acknowledged. The single-center design may limit generalizability, and while the sample was adequate for Rasch analysis, 65% of participants had RA, with fewer cases of axSpA and PsA. Consequently, findings may not represent all IA subtypes. Disease characteristics and work challenges vary across conditions, so future studies should evaluate the CIJSS separately within each group using larger, more diverse samples. The study included only individuals in paid employment, excluding unpaid workers such as caregivers or homemakers, who may also experience significant work-related stress. As disease characteristics and work-related challenges can vary across diagnostic groups, future research should assess the validity and reliability of the CIJSS separately within each subgroup using larger and more diverse samples. This would strengthen the evidence base for the scale’s use across the full spectrum of IA.

Common comorbidities such as OA, obesity, and fibromyalgia may also contribute to perceived job strain independent of disease activity. Although associations between pain, fatigue, mobility limitations, and CIJSS scores were explored, comorbidities could introduce construct-irrelevant variance. Future studies should account for comorbidities to improve measurement precision and further validate the scale across the full spectrum of IA.

## Conclusions

This study demonstrates that the Turkish version of the CIJSS is a valid, reliable, and culturally appropriate measure of job strain in people with IA (RA, axSpA, and PsA). It is suitable for use in clinical and research settings to evaluate work-related challenges and inform tailored interventions.

## Supplementary Material

rkaf142_Supplementary_Data

## Data Availability

The data set of this study can be requested from the corresponding author for a reasonable reason. The Turkish CIJSS is available in Supplementary File S3, available at *Rheumatology Advances in Practice* Online.
