# Supplementary material for: Translation, validity and reliability of the Turkish Chronic Illness Job Strain Scale (CIJSS) in people with inflammatory arthritis
Source: Rheumatol Adv Pract. 2025 Dec 2;10(1):rkaf142. doi: 10.1093/rap/rkaf142 (PMC12758117; doi:10.1093/rap/rkaf142)
Supplement: rkaf142_Supplementary_Data [file rkaf142_supplementary_data.zip › Suppl_File_3.Kronik_Hastalik_I?s,_Zorlanma_O"lc,eg?i.docx]

**KRONİK HASTALIK İŞ ZORLANMA ÖLÇEĞİ**

Bu ölçeğin amacı kronik hastalıklardaki iş yükünün işin çeşitli yönleriyle değerlendirilmesidir. Öğelerdeki "artrit" kelimesi başka koşullarla (örn. multipl skleroz) değiştirilebilir.

**References**:

Gignac, M.A.M., Sutton, D., & Badley, E.M. (2007). Arthritis symptoms, the work environment, and the future: Measuring perceived job strain among employed people with arthritis. Arthritis Care & Research, 57, 738-747. [**Pub Med ID** 17530672]

Gignac, M.A.M. & Cao, X. (2009). “Should I tell my employer and co-workers I have arthritis?”: A longitudinal examination of self-disclosure in the workplace. Arthritis Care & Research, 61, 1753-1761.

**Puanlama:** On beş madde üzerinden ortalama bir puan hesaplanabilir veya puanlar toplam bir puan elde etmek için toplanabilir. Ortalama puanlar kullanılıyorsa, eksik veriler için bireyin ortalama puanının atfedildiği 3 maddeye kadar eksik verilere izin verilir.

**Açıklamalar:** Artrit hastası olmayı ve istihdam edilmeyi stresli hale getirebilecek bir dizi faktör vardır. Aşağıdaki sorularla artrit hastası olmanın çalışmanızı sizin için ne ölçüde stresli hale getirdiğini öğrenmek istiyoruz:

**Kronik Hastalık İş Zorlanma Ölçeği**

Artrit hastası olmayı ve istihdamı stresli hale getirebilecek bir dizi faktör vardır. Aşağıdaki sorularla artrit hastası olmanın çalışmanızı sizin için ne ölçüde stresli hale getirdiğini öğrenmek istiyoruz:

|  | Hiç Stresli Değil | Az stresli | Biraz Stresli | Oldukça Stresli | Son Derece Stresli |
| --- | --- | --- | --- | --- | --- |
| 1.Artritle ilgili şikâyetleriniz (örneğin, ağrı, yorgunluk, vb.) işinizi ne kadar stresli hale getiriyor? | 111 1 | 2 | 3 | 4 | 5 |
| 2. İş yerinde günden güne nasıl hissedeceğinize dair belirsizlik ne kadar streslidir? | 111 1 | 2 | 3 | 4 | 5 |
| 3. Artritinizle birlikte işteki programınız işinizi ne kadar stresli hale getiriyor? | 111 1 | 2 | 3 | 4 | 5 |
| 4. Artritinizle birlikte işinizin gerekleri işinizi ne kadar stresli hale getiriyor? | 111 1 | 2 | 3 | 4 | 5 |
| 5. Artritin şu anda veya gelecekte mali durumunuz üzerindeki etkisi hakkındaki düşünceler ne kadar streslidir? | 111 1 | 2 | 3 | 4 | 5 |
| 6. Artritle ilgili işe devamsızlığı yönetmek ne kadar streslidir? | 111 1 | 2 | 3 | 4 | 5 |
| 7. Artritin ve işin nasıl idare edileceğine ilişkin bilgi ve/veya kaynakların eksikliği ne kadar streslidir? | 111 1 | 2 | 3 | 4 | 5 |
| 8. Artritin işinizde kalma beceriniz üzerindeki etkisi hakkındaki düşünceler ne kadar streslidir? | 111 1 | 2 | 3 | 4 | 5 |
| 9. Artritin yeteneklerinizi kullanma beceriniz ve eğitiminiz üzerindeki etkisi işinizi ne kadar stresli hale getiriyor? (yani, eğitiminizi kullanamamak) | 111 1 | 2 | 3 | 4 | 5 |
| 10. Artritinizin gelecekteki kariyer planlarınız üzerindeki etkisi hakkındaki düşünceler ne kadar streslidir? | 111 1 | 2 | 3 | 4 | 5 |
| 11. Artritiniz dikkate alındığında, işvereninizle şu anki ilişkiniz veya işvereninizle gelecekteki ilişkinize ilişkin endişeleriniz ne kadar streslidir? | 111 1 | 2 | 3 | 4 | 5 |
| 12. Artritiniz dikkate alındığında, iş arkadaşlarınızla şu anki ilişkiniz veya iş arkadaşlarınızla gelecekteki ilişkinize ilişkin endişeleriniz ne kadar streslidir? | 111 1 | 2 | 3 | 4 | 5 |
| 13.Artritinizin “görünmezliği” işinizi ne kadar stresli hale getiriyor? (bununla insanların günden güne nasıl hissettiğinizi anlayamadıklarını kastediyorum) | 111 1 | 2 | 3 | 4 | 5 |
| 14. Sağlığınızı, işinizi ve özel yaşamınızı dengelemek ne kadar streslidir? | 111 1 | 2 | 3 | 4 | 5 |
| 15. Artritinizin bir sonucu olarak yaşamınızda meydana gelen değişiklikleri (örn. koşullardaki değişiklikler, kimlik duygunuz vb.) kabul etmeye çalışırken ne kadar stres yaşadınız? | 111 1 | 2 | 3 | 4 | 5 |
