# Supplementary material for: Translation, validity and reliability of the Turkish Chronic Illness Job Strain Scale (CIJSS) in people with inflammatory arthritis
Source: Rheumatol Adv Pract. 2025 Dec 2;10(1):rkaf142. doi: 10.1093/rap/rkaf142 (PMC12758117; doi:10.1093/rap/rkaf142)
Supplement: rkaf142_Supplementary_Data [file rkaf142_supplementary_data.zip › Suppl_File_5._Person-Item_Threshold_Distribution_.docx]

**Supplementary File S5.** Person-Item Threshold Distribution


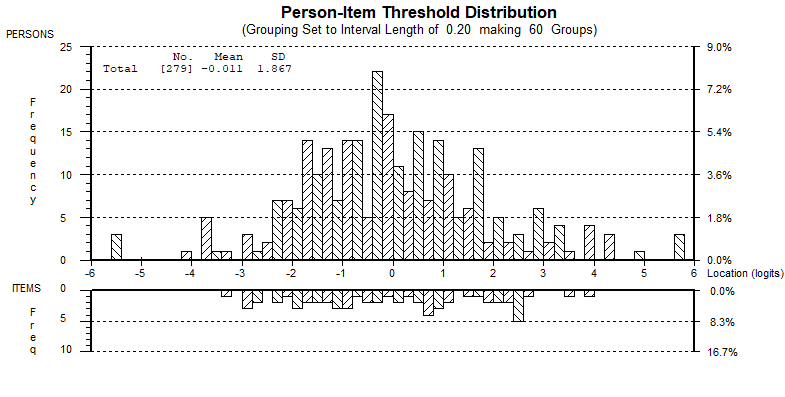


**Suppl File 5 text.**  Person–item threshold distribution for the Turkish CIJSS. The upper panel shows the distribution of person measures and the lower panel displays item thresholds on the same logit scale, demonstrating the alignment between the scale’s item difficulty and participant ability levels.
