# Supplementary material for: Translation, validity and reliability of the Turkish Chronic Illness Job Strain Scale (CIJSS) in people with inflammatory arthritis
Source: Rheumatol Adv Pract. 2025 Dec 2;10(1):rkaf142. doi: 10.1093/rap/rkaf142 (PMC12758117; doi:10.1093/rap/rkaf142)
Supplement: rkaf142_Supplementary_Data [file rkaf142_supplementary_data.zip › Suppl_File_1.COSMIN_checklist__CIJSS.docx]

**Supplementary File S1. COSMIN CHECKLIST**

**Box 1.** PROM development

* This is an adaptation of an existing instrument, with no original scale development

| **1a. Concept elicitation study (relevance and comprehensiveness)** | | | | | | | | | | | | | | | | | | | | | | | | |  |  |  |  |
| --- | --- | --- | --- | --- | --- | --- | --- | --- | --- | --- | --- | --- | --- | --- | --- | --- | --- | --- | --- | --- | --- | --- | --- | --- | --- | --- | --- | --- |
| 1 | | Was the concept elicitation study performed in a sample representing the target population for which the PROM was developed? | | | | | |  | | | |  | | | |  |  | | | | | | | N/A |  |  |  |  |
| 2 | | Was an appropriate qualitative data collection method used to identify relevant items for a new PROM? | | | | | |  | | | |  | | | |  |  | | | | | | | N/A |  |  |  |  |
| 3 | | Were skilled group moderators/interviewers used? | | | | | |  | | | |  | | | |  |  | | | | | | | N/A |  |  |  |  |
| 4 | | Were the group meetings or interviews based on an appropriate topic or interview guide? | | | | | |  | | | |  | | | |  |  | | | | | | | N/A |  |  |  |  |
| 5 | | Were the group meetings or interviews recorded and transcribed verbatim? | | | | | |  | | | |  | | | |  |  | | | | | | | N/A |  |  |  |  |
| 6 | | Was an appropriate approach used to analyse the data | | | | | |  | | | |  | | | |  |  | | | | | | | N/A |  |  |  |  |
| 7 | | Was at least part of the data coded independently? | | | | | |  | | | |  | | | |  | N/A | | | | | | | |  |  |  |  |
| 8  9 | | | | Was data collection continued until saturation was reached? | |  | | | |  | | | |  | | | | |  | | | N/A | | | | | | |
|  |  |  |  | For quantitative studies (surveys): was the sample size appropriate? | |  | | | |  | | | |  | | | | |  | | | N/A | | | | | | |
| 10 | | | | Were there any other important flaws in the design or methods of the study? | |  | | | |  | | | |  | | | | |  | | | N/A | | | | | | |
| **1b. Pilot study (Cognitive interview study or other pilot test)** (comprehensibility) | | | | | | | | | | | | | | | | | | | | | | | | | | | | |
| 11 | | | | Was the pilot study performed in a sample representing the target population for which the PROM was developed? | |  | | | |  | | | |  | | | | |  | | | N/A | | | | | | |
| 12 | | | | Was the comprehensibility assessed of the PROM instructions, items, response options, and recall period? | |  | | | |  | | | |  | | | | |  | | | Yes | | | | | | |
| 13 | | | | Were all items tested in their final form? | |  | | | |  | | | |  | | | | |  | | | Yes | | | | | | |
| 14 Was an appropriate qualitative method used? | | | |  | | | |  | | | |  | | | | |  | | | Yes | | | | | | |  |  |
| 15. Was each item tested in an appropriate number of patients? For qualitative studies For quantitative (survey) studies | | | |  | | | |  | | | |  | | | | |  | | | Yes | | | | | | |  |  |
| 16. Were skilled interviewers used? | | | |  | | | |  | | | |  | | | | |  | | | Yes | | | | | | |  |  |
| 17. Were the interviews based on an appropriate interview guide? | | | |  | | | |  | | | |  | | | | |  | | | Yes | | | | |  |  |  |  |
| 18. Were the interviews recorded and transcribed verbatim? | | | |  | | | |  | | | |  | | | | |  | | | Yes | | | | |  |  |  |  |
| 19. | | | Was an appropriate approach used to analyze the data? | | | |  | | | |  | | | |  | | | | |  | | | Yes | | | |  |  |
|  | | |  |  |  |  |  | | | |  | | | |  | | | | |  | | |  | | | |  |  |
| 20. | | | Were at least two researchers involved in the analysis? | | | |  | | | |  | | | |  | | | | |  | | | Yes | | | |  |  |
| 21. | | | Were problems regarding the comprehensibility of the PROM instructions, items, response options, and recall period appropriately addressed by adapting the PROM? | | | |  | | | |  | | | |  | | | | |  | | | Yes | | | |  |  |
| 22. | | | Were there any other important flaws in the design or methods of the study? | | | |  | | | |  | | | |  | | | | |  | | | No | | | |  |  |

**Box 2. Content validity**

| **2a. Asking patients about relevance** | | | | | | | | | | | | | | | | | | | | | | | | | | | | | | | | | |  |  |  |
| --- | --- | --- | --- | --- | --- | --- | --- | --- | --- | --- | --- | --- | --- | --- | --- | --- | --- | --- | --- | --- | --- | --- | --- | --- | --- | --- | --- | --- | --- | --- | --- | --- | --- | --- | --- | --- |
| *Design requirements* | | | | | | | |  | | | | |  | | | | | |  | | | | | | |  | | |  | | | | |  |  |  |
| 1 | Was an appropriate method used to ask patients whether each item is relevant for their experience with the condition? | | | | | | |  | | | | |  | | | | | |  | | | | | | |  | | | Yes | | | | |  |  |  |
| 2 | Was each item tested in an appropriate number of patients? For qualitative studies For quantitative (survey) studies | | | | | | |  | | | | |  | | | | | |  | | | | | | |  | | |  | | | | |  |  |  |
|  |  |  |  |  |  |  |  |  | | | | |  | | | | | |  | | | | | | |  | | | Yes  Cognitive debriefing interviews n=30 | | | | |  |  |  |
| 3 | Were skilled group moderators/interviewers used? | | | | | | |  | | | | |  | | | | | |  | | | | | | |  | | | Yes | | | | |  |  |  |
| 4 | Were the group meetings or interviews based on an appropriate topic or interview guide? | | | | | | |  | | | | |  | | | | | |  | | | | | | |  | | | Yes  Yes | | | | |  |  |  |
| 5 | Were the group meetings or interviews recorded and  transcribed verbatim? | | | | | | |  | | | | |  | | | | | |  | | | | | | |  | | |  | | | | |  |  |  |
| *Analyses* | | | | | | | | | | | | | | | | | | | | | | | | | | | | | | | | |  |  |  |  |
| 6 | | | | Was an appropriate approach used to analyse the data? | |  | | | | | | | |  | | | | | |  | | | |  | | | | Yes | | | | |  |  |  |  |
| 7 | | | | Were at least two researchers involved in the analysis? | |  | | | | | | | |  | | | | | |  | | | |  | | | | Yes | | | | |  |  |  |  |
| 8 | | | | Were there any other important flaws in the design or methods of the study? | |  | | | | | | | |  | | | | | |  | | | |  | | | | No | | | | |  |  |  |  |
| 2b. **Asking patients about comprehensiveness** | | | | | | | | | | | | | | | | | | | | | | | | | | | | | | | | |  |  |  |  |
| *Design requirements* | | | | | | | | | | | | | | | | | | | | | | | | | | | | | | | | |  |  |  |  |
| 9 | | | | Was an appropriate method used for assessing the comprehensiveness of the PROM? | |  | | | | | | | |  | | | | | |  | | | | In the cognitive debriefing phase, comprehensiveness was assessed as follows: not easy to complete, partly easy and easy. Relevance was assessed as very much, a lot, partly and not relevant. | | | | | | | |  |  |  |  |  |
| 10 | | | | Was the PROM tested in an appropriate number of patients?  For qualitative studies  For quantitative (survey) studies | |  | | | | | | | |  | | | | | |  | | | |  | | | |  | | | | |  |  |  |  |
|  | | | |  |  |  | | | | | | | |  | | | | | |  | | | | Yes n=30. | | | |  | | | | |  |  |  |  |
| 11 | | | | Were skilled group moderators/interviewers used? | |  | | | | | | | |  | | | | | |  | | | | Yes | | | |  | | | | |  |  |  |  |
| 12 | | | | Were the group meetings or interviews based on an appropriate topic or interview guide? | |  | | | | | | | |  | | | | | |  | | | | Yes | | | |  | | | | |  |  |  |  |
| 13 | | | Were the group meetings or interviews recorded and transcribed verbatim? | | | | Yes | | | |  | | | | | | |  | | | | | | | | |  | | |  | | | | | |  |
| *Analyses* | | | | | | |  | | | |  | | | | | | |  | | | | | | | | |  | | |  | | | | | |  |
| 14 | | | Was an appropriate approach used to analyse the data? | | | |  | | | |  | | | | | | |  | | | | | | | | |  | | | Yes | | | | | |  |
| 15 | | | Were at least two researchers involved in the analysis? | | | |  | | | |  | | | | | | |  | | | | | | | | |  | | | Yes | | | | | |  |
| 16 | | | Were there any other important flaws in the design or methods of the study? | | | |  | | | |  | | | | | | |  | | | | | | | | |  | | | No | | | | | |  |
| **2c. Asking patients about comprehensibility** | | | | | | | | | | |  | | | | | | |  | | | | | | | | |  | | |  | | | | | |  |
| 17 | | | Was an appropriate qualitative method used for assessing the comprehensibility of the PROM instructions, items, response options, and recall period? | | | |  | | | |  | | | | | | |  | | | | In the cognitive debriefing phase, comprehensiveness was assessed as follows: not easy to complete, partly easy and easy. Relevance was assessed as very much, a lot, partly and not relevant. | | | | | | | |  | | | | | |  |
| 18 | | | Was each item tested in an appropriate number of patients?  For qualitative studies  For quantitative (survey) studies | | | |  | | | |  | | | | | | |  | | | | | | | | |  | | |  | | | | | |  |
|  | | |  |  |  |  |  | | | |  | | | | | | |  | | | | | | | | | Yes n=30. | | |  | | | | | |  |
| 19 | | Were skilled group moderators/interviewers used? | | |  | | | | |  | |  | | | | |  | | | | | | Yes | | | | | | | |  | | | |  |  |
| 20 | | Were the group meetings or interviews based on an appropriate topic or interview guide? | | |  | | | | | | |  | | | | |  | | | | | | Yes | | | | | | | |  | | | |  |  |
| 21 | | Were the group meetings or interviews recorded and transcribed verbatim? | | |  | | | | | | |  | | | | |  | | | | | | Yes | | | | | | | |  | | | |  |  |
| Analyses | | | | | | | | | | | | | | | | | | | | | | | | | | | | | | | | | | |  |  |
| 22 | | Was an appropriate approach used to analyse the data? | | |  | | | | |  | |  | | | | |  | | | | | | Yes | | | | | | | |  | | | |  |  |
| 23 | | Were at least two researchers involved in the analysis? | | |  | | | | |  | |  | | | | |  | | | | | | Yes | | | | | | | |  | | | |  |  |
| 24 | | Were there any other important flaws in the design or methods of the study? | | |  | | | | |  | |  | | | | |  | | | | | | No | | | | | | | |  | | | |  |  |
| **2d.** **Asking professionals about relevance** | | | | | | | | | | | | | | | | | | | | | | | | | | | | | | | | | | |  |  |
| *Design requirements* | | | | |  | | | | |  | | | | | |  |  | | | | | |  | | | | | | | |  | | | |  |  |
| 25 | | Was an appropriate method used to ask professionals whether each item is relevant for the construct of interest? | | |  | | | | |  | | | | | |  | | | | | | | Yes | | | | | | | |  | | | |  |  |
| 26 Were professionals from all relevant disciplines included? | | | | | | | | |  | | | | | |  | | | | | |  | | | | Yes | | | | | | | | | | | |
|  |  |  |  |  |  |  |  |  |  | | | | | |  |  |  |  |  |  |  | | | |  | | | | | | | | | | | |
|  |  |  |  |  |  |  |  |  |  | | | | | |  |  |  |  |  |  |  | | | |  | | | | | | | | | | | |
| 27 Was each item tested in an appropriate number of professionals?  For qualitative studies  For quantitative (survey) studies | | | | | | | | |  | | | | | |  | | | | | |  | | | | Yes | | | | | | | | | | | |
|  |  |  |  |  |  |  |  |  |  | | | | | |  | | | | | |  | | | |  | | | | | | | | | | | |
|  |  |  |  |  |  |  |  |  |  | | | | | |  |  |  |  |  |  |  | | | |  | | | | | | | | | | | |
|  |  |  |  |  |  |  |  |  |  |  |  |  |  |  |  |  |  |  |  |  |  |  |  |  |  | | | | | | | | | | | |
| *Analyses* | | | | | | | | |  | | | | | |  | | | | | |  | | | |  | | | | | | | | | | | |
| 28 Was an appropriate approach used to analyse the data? | | | | | | | | |  | | | | | | | | | | | |  | | | | Yes | | | | | | | | | | | |
|  |  |  |  |  |  |  |  |  |  |  |  |  |  |  |  |  |  |  |  |  |  | | | |  | | | | | | | | | | | |
|  | | | | | | | | |  |  |  |  |  |  |  |  |  |  |  |  |  | | | |  | | | | | | | | | | | |
| 29 Were at least two researchers involved in the analysis? | | | | | | | | |  | | | | | |  | | | | | |  | | | | Yes | | | | | | | | | | | |
|  |  |  |  |  |  |  |  |  |  | | | | | |  |  |  |  |  |  |  |  |  |  |  | | | | | | | | | | | |
|  | | | | | | | | |  | | | | | |  |  |  |  |  |  |  |  |  |  |  | | | | | | | | | | | |
| 30 Were there any other important flaws in the design or methods of the study? | | | | | | | | |  | | | | | |  | | | | | |  | | | | Yes | | | | | | | | | | | |
| **2e. Asking professionals about comprehensiveness**  *Design requirement* | | | | | | | | | | | | | | | | | | | | | | | | | | | | | | | | | | | | |
| 31 Was an appropriate method used for ass essing the comprehensiveness of the PROM? | | | | | | | | |  | | | | | |  | | | | | |  | | | | Comprehensiveness was assessed as follows: not easy to complete, partly easy and easy. Relevance was assessed as very much, a lot, partly and not relevant. | | | | | | | | | | | |
|  |  |  |  |  |  |  |  |  |  |  |  |  |  |  |  |  |  |  |  |  |  | | | |  |  |  |  |  |  |  |  |  |  |  |  |
|  |  |  |  |  |  |  |  |  |  |  |  |  |  |  |  |  |  |  |  |  |  | | | |  |  |  |  |  |  |  |  |  |  |  |  |
|  | | | | | | | | |  | | | | | |  |  |  |  |  |  |  | | | |  | | | | | | | | | | | |
| 32.Were professionals from all relevant disciplines included? | | | | | | | | |  | | | | | |  | | | | | |  | | | | Yes | | | | | | | | | | | |
|  |  |  |  |  |  |  |  |  |  |  |  |  |  |  |  |  |  |  |  |  |  |  |  |  |  | | | | | | | | | | | |
|  | | | | | | | | |  | | | | | |  |  |  |  |  |  |  |  |  |  |  | | | | | | | | | | | |
| 33. Was the PROM tested in an appropriate number of professionals?  For qualitative studies  For quantitative (survey) studies | | | | | | | | |  | | | | | |  | | | | | |  | | | |  | | | | | | | | | | | |
|  |  |  |  |  |  |  |  |  |  | | | | | |  | | | | | |  | | | | Yes | | | | | | | | | | | |
| Analyses | | | | | | | | |  | | | | | |  | | | | | |  | | | |  | | | | | | | | | | | |
| 34. Was an appropriate approach used to analyse the data? | | | | | | | | |  | | | | | |  | | | | | |  | | | | Yes | | | | | | | | | | | |
|  | | | | | | | | |  |  |  |  |  |  |  |  |  |  |  |  |  |  |  |  |  |  |  |  |  |  |  |  |  |  |  |  |
| 35. Were at least two researchers involved in the analysis? | | | | | | | | |  | | | | | |  | | | | | |  | | | | Yes | | | | | | | | | | | |
|  |  |  |  |  |  |  |  |  |  |  |  |  |  |  |  |  |  |  |  |  |  |  |  |  |  | | | | | | | | | | | |
|  | | | | | | | | |  | | | | | |  |  |  |  |  |  |  |  |  |  |  | | | | | | | | | | | |
| 36. Were there any other important flaws in the design or methods of the study? | | | | | | | | |  | | | | | |  | | | | | |  | | | | No | | | | | | | | | | | |
| **2f. Asking professionals about comprehensibility** | | | | | | | | | | | | | | | | | | | | | | | | | | | | | | | | | | | | |
| *Design requirement* | | | | | | | | |  | | | | | |  | | | | | |  | | | |  | | | | | | | | | | | |
| 37. Was an appropriate method used for assessing the comprehensibility of the PROM instructions, items, response options, and recall period? | | | | | | | | |  | | | | | |  | | | | | |  | | | | Yes | | | | | | | | | | | |
| 38. Were professionals from all relevant disciplines included? | | | | | | | | |  | | | | | |  | | | | | |  | | | | Yes | | | | | | | | | | | |
| 39. Was each item tested in an appropriate number of professionals?  For qualitative studies  For quantitative (survey) studies | | | | | | | | |  | | | | | |  | | | | | |  | | | | Yes | | | | | | | | | | | |
| *Analyses* | | | | | | | | |  | | | | | |  | | | | | |  | | | |  | | | | | | | | | | | |
| 40. Was an appropriate approach used to analyse the data? | | | | | | | | |  | | | | | |  | | | | | |  | | | | Yes | | | | | | | | | | | |
| 41. Were at least two researchers involved in the analysis? | | | | | | | | |  | | | | | |  | | | | | |  | | | | Yes | | | | | | | | | | | |
| 42. Were there any other important flaws in the design or methods of the study? | | | | | | | | |  | | | | | |  | | | | | |  | | | | No | | | | | | | | | | | |

**Box 3.** Structural validity

| *Statistical methods* | |  |  |  |  |  | | |
| --- | --- | --- | --- | --- | --- | --- | --- | --- |
| 1 | For CTT: Was exploratory or confirmatory factor analysis performed? |  |  |  | N/A |  | | |
| 2 | For IRT/Rasch: does the chosen model fit to the research question? |  |  |  | Rasch analysis was appropriately used to assess the unidimensional structure and item fit of the CIJSS. Advanced techniques such as super-item and bi-factor modeling were applied. | |  | |
| 3 | Was the sample size included in the analysis adequate? |  |  |  | For Rasch analysis, ≥200 people are required. A sample of 200 people was used in the study. | | |  |
| *Other* | |  |  |  |  |  | | |
| 4 | Were there any other important flaws in the design or statistical methods of the study? |  |  |  | Appropriate statistical methods (Rasch analysis, superitem analysis, DIF analysis, etc.) were used in detail, and no significant methodological errors were reported. | |  | |

**Box 4. Internal consistency**

| *Statistical methods* | |  |  |  |  |  | | | |
| --- | --- | --- | --- | --- | --- | --- | --- | --- | --- |
| 1 | For continuous scores: Was Cronbach’s alpha or omega calculated? |  |  | | Cronbach alpha coefficient was calculated and reported (α = 0.96) | | | |  |
| 2 | For dichotomous scores: Was Cronbach’s alpha or KR-20 calculated? |  |  | | Not applicable. The scale has a Likert type (1–5) scoring system; dichotomous data was not used. | | |  | |
| 3 | For IRT-based scores: Was standard error of the theta (SE (θ)) or reliability coefficient of estimated latent trait value (index of (subject or item) separation) calculated? |  |  |  | In Rasch analysis, person separation index and variance explanation ratio are reported, but SE (θ) is not stated directly. | |  | | |
|  | *Other* |  |  |  |  |  | | | |
| 4 | Were there any other important flaws in the design or statistical methods of the study? |  |  | | No |  | | | |

**Box 5. Cross-cultural validity\Measurement invariance**

| *Design requirements* | |  |  |  |  | |  |
| --- | --- | --- | --- | --- | --- | --- | --- |
| 1 | Were the samples similar for relevant characteristics except for the group variable? |  | . | The Turkish version and the original English version were compared and DIF analysis was performed | |  |  |
| *Statistical methods* | | | | | | | |
| 2 | Was an appropriate approach used to analyse the data? |  |  | DIF (Differential Item Functioning) analyses were performed within the Rasch model, and measurement invariance was tested across samples. A comparison was made between UK and Turkish data. | |  |  |
| 3 | Was the sample size included in the analysis adequate? |  |  | For Rasch analysis, ≥200 people are required. A sample of 200 people was used in the study. | |  |  |
|  | Other |  |  |  |  | |  |
| 4 | Were there any other important flaws in the design or statistical methods of the study? |  |  | The analysis method was appropriate, descriptive and cross-cultural comparison was made. DIF findings were reported, no significant error was noted. | |  |  |

**Box 6. Reliability**

| *Design requirements* | |  |  |  | | | | | |  | | |  |
| --- | --- | --- | --- | --- | --- | --- | --- | --- | --- | --- | --- | --- | --- |
| 1 | Were patients stable on the construct to be measured in the time between the repeated measurements? |  |  | | Participants were assumed to be stable, but symptom change was not directly assessed. | | | | | | |  |  |
| 2 | Was the time interval between the repeated measurements appropriate? |  |  | The test-retest interval was 2 weeks. | | | | | | | |  |  |
| 3 | Were the measurement conditions similar for the repeated measurements – _except for the condition being evaluated? |  |  | Yes | | | | | |  | | |  |
| *Statistical methods* | | | | | | | | | | | | | |
| 4 | For continuous scores: Was the appropriate intraclass correlation coefficient (ICC) calculated? |  |  | | | | ICC (2,1) = 0.88, Spearman correlation (r = 0.886) is also given | | | | |  |  |
| 5 | For dichotomous scores: was kappa calculated? |  |  | | | Not Applicable. The scale is not ordinal, but Likert type continuous scores are used | | | | | |  | . |
| 6 | For nominal scores: was an unweighted kappa calculated? |  |  | | | | | Not applicable. Kappa coefficient was not calculated because it was not required. | | | |  |  |
| 7 | For ordinal scores: was a weighted kappa calculated? |  |  | | | | | | Not applicable. No ordinal structure to require weighting. | |  | |  |
| *Other* |  |  |  |  | | | | | |  | | |  |
| 8 | Were there any other important flaws in the design or statistical methods of the study? |  |  | No. | | | | | |  | | |  |

**Box 7.** Measurement error

| *Design requirements* | |  |  |  |  |  | |
| --- | --- | --- | --- | --- | --- | --- | --- |
| 1 | Were patients stable on the construct to be measured in the time between the repeated measurements? |  |  |  | Stability was assumed, but symptom levels were not directly measured. | |  |
| 2 | Was the time interval between the repeated measurements appropriate? |  |  |  | Time interval was two weeks. |  | |
| 3 | Were the measurement conditions similar for the measurements – _except for the condition being evaluated as a source of variation? |  |  |  | Yes |  | |
| *Statistical methods* | | | | | | | |
| 4 | For continuous scores: was the Standard Error of Measurement (SEM), Smallest Detectable Change (SDC) or Limits of Agreement (LoA) calculated? |  |  |  | SEM (3.65) and MDC (5.30) were calculated. |  | |
| 5 | For dichotomous/nominal/ordinal scores: was the percentage (positive and negative) agreement calculated? |  |  |  | Not applicable. Percent agreement is unnecessary since scores are on a continuous scale. |  | |
| Other | |  |  |  |  |  | |
| 6 | Were there any other important flaws in the design or statistical methods of the study? |  |  |  | No. |  | |

**Box 8.** Criterion validity

| *Statistical methods* | |  |  |  | |  | |  |
| --- | --- | --- | --- | --- | --- | --- | --- | --- |
| 1 | For continuous scores: were correlations, or the AUC calculated? |  |  | | Correlations were calculated between the CIJSS and WLQ, WPAI, RAID and HAQ | |  |  |
| 2 | For dichotomous scores: were sensitivity and specificity determined? |  |  | | Not applicable. There is no dichotomous result/gold standard in the scale. | |  |  |
| Other | |  |  |  | |  | |  |
| 3 | Were there any other important flaws in the design or statistical methods of the study? |  |  | No. | |  | |  |

**Box 9.** Hypotheses testing for construct validity

| **9a. Comparison with other outcome measurement instruments (convergent validity)** | | | | | | | | | | | | | | | |  |
| --- | --- | --- | --- | --- | --- | --- | --- | --- | --- | --- | --- | --- | --- | --- | --- | --- |
| *Design requirements* | | |  | |  | |  | |  | | | | |  | |  |
| 1 | | Is it clear what the comparator instrument(s) measure(s)? |  | |  | |  | | All compared tools (WLQ, WPAI, HAQ, RAID) clearly define what they measure. | | | | |  | |  |
| 2 | | Were the measurement properties of the comparator instrument(s) sufficient? |  | |  | |  | | The compared scales are adapted into Turkish and their validity and reliability have been demonstrated. | | | | |  | |  |
| *Statistical methods* | | |  | |  | |  | |  | | | | |  | |  |
| 3 | | Were statistical methods adequate for the comparisons made? |  | |  | |  | | Yes. Construct validity was assessed using Rasch analysis. | | | | |  | |  |
| Other | | |  | |  | |  | |  | | | | |  | |  |
| 4 | | | Were there any other important flaws in the design or statistical methods of the study? |  | |  | |  | | No. | | | | |  | |
| 9b. **Comparison between subgroups (discriminative or known-groups validity)** | | | | | | | | | | | | | |  |  |  |
| *Design requirements* | | |  | |  | |  | | |  | |  | |  |  |  |
| 5 | Was an adequate description provided of important characteristics of the subgroups? | |  | |  | | | |  | NA |  | | |  |  |  |
| *Statistical methods* | | |  | |  | |  | | |  | |  | |  |  |  |
| 6 | Were statistical methods appropriate for the subgroups being compared? | |  | |  | |  | | | Yes | |  | |  |  |  |
| Other | | |  | |  | |  | | |  | |  | |  |  |  |
| 7 | Were there any other important flaws in the design or statistical methods of the study? | |  | |  | |  | | | No | |  | |  |  |  |

**Box 10.** Responsiveness

| **10a. Criterion approach (i.e. comparison to a gold standard)** | | | | | | |
| --- | --- | --- | --- | --- | --- | --- |
| *Statistical methods* | |  |  |  |  |  |
| 1 | For continuous scores: were correlations between change scores, or the AUC calculated? |  |  |  | Correlation with ROC curve or change scores is reported. |  |
| 2 | . For dichotomous scales: were sensitivity and specificity (changed versus not changed) determined? |  |  |  | Not applicable. No dichotomous measurement. |  |
| *Other* | |  |  |  |  |  |
| 3 | Were there any other important flaws in the design or statistical methods of the study? |  |  |  | No. |  |
