# Supplementary material for: Translation, validity and reliability of the Turkish Chronic Illness Job Strain Scale (CIJSS) in people with inflammatory arthritis
Source: Rheumatol Adv Pract. 2025 Dec 2;10(1):rkaf142. doi: 10.1093/rap/rkaf142 (PMC12758117; doi:10.1093/rap/rkaf142)
Supplement: rkaf142_Supplementary_Data [file rkaf142_supplementary_data.zip › Suppl_File_4._Threshold_ordering.docx]

**Supplementary File S4.** Threshold ordering


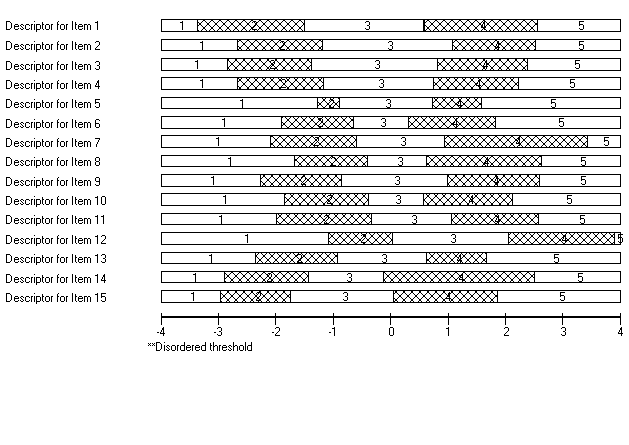


**Suppl File 4 Alt text.** Category threshold map showing the response category thresholds for the 15 items of the Turkish Chronic Illness Job Strain Scale (CIJSS). The figure illustrates the ordering of thresholds across items, identifying items with disordered thresholds based on Rasch analysis.
