# Supplementary material for: Translation, validity and reliability of the Turkish Chronic Illness Job Strain Scale (CIJSS) in people with inflammatory arthritis
Source: Rheumatol Adv Pract. 2025 Dec 2;10(1):rkaf142. doi: 10.1093/rap/rkaf142 (PMC12758117; doi:10.1093/rap/rkaf142)
Supplement: rkaf142_Supplementary_Data [file rkaf142_supplementary_data.zip › Suppl_File_2.Rasch_measurement.docx]

**Translation, Validity, and Reliability of the Turkish Version of the Chronic Illness Job Strain Scale (CIJSS) in People with Inflammatory Arthritis**

**Supplementary File S2**

***Statistical Analysis***

*Rasch Model:*

The scale was tested against the requirements of the Rasch measurement Model using the partial credit parametrization (1, 2). Briefly this requires satisfactory levels of unidimensionality, local item independence, invariant probabilistic ordering of items (homogeneity), monotonicity of polytomous items (i.e. threshold ordering) and adequate reliability for the intended purpose (i.e. group- or individual- level analysis). Full details of these requirements, and levels of acceptability can be found in Tennant A, Küçükdeveci AA (2023) (3).

Invariance was tested through Differential Item Functioning (DIF) of contextual factors, which included time, age, sex, diagnosis, medication, Body Mass Index (BMI) and duration (4) was tested by threshold ordering, and homogeneity through examination of the probabilistic ordering of items with Chi-Square fit statistics. Local Item Independence was tested through correlations within the item residuals (5). Where this test failed, consideration was given to grouping locally dependent items into “super-items” by simply adding them together (6). If a conceptually based grouping is also possible, then testlets can be derived and, if the analysis results in two testlets, a conditional Chi-Square fit statistic becomes available (7). Unidimensionality is tested though Smith’s t-test of residuals (8).

Should fit be achieved, then the result is tested for invariance (by DIF) against the British English version (9, 10). Should invariance be achieved then a comparison between countries will be valid. Else, information may be gained about where such lack of invariance is found, providing information back to the developers.

***Results***

## *Rasch Analysis of the Chronic Illness Job Strain Scale (CIJSS)*

Various pairs of locally dependent items were evident (Table 1). For example, items 3 and 4 “How Stressful do your shifts or work hours, combined with your condition, make your work?” and “How stressful do the demands of your job, combined with your condition, make your work?”, had a residual correlation of 0.740, indicating almost identical items. In all, there were eleven pairs of locally dependent items grouped into four clusters.

The transformation table of raw score to the metric CIJSS from this solution is given in Table 2. This sets the item range as 0-4. If data were entered as 1-5, add 15 to the raw score in the table. The metric remains unchanged and ranged from 0-60. This seems more appropriate to, for example, indicate the absence of job strain by a score of zero, rather than 15.

The data were then tested for cross-cultural invariance against the English version if the scale using the final solution above. Fit was good. It was unidimensional, and invariant across culture (DIF).

**Table 1.** Residual Correlation Pattern

Item I0001 I0002 I0003 I0004 I0005 I0006 I0007 I0008 I0009 I0010 I0011 I0012 I0013 I0014 I0015

I0001 I0001 1.000
I0002 I0002 **0.251** 1.000
I0003 I0003 **0.381** **0.307** 1.000
I0004 I0004 **0.427** **0.241** **0.740** 1.000
I0005 I0005 -0.163 -0.335 -0.323 -0.282 1.000
I0006 I0006 -0.231 -0.041 -0.207 -0.192 0.113 1.000
I0007 I0007 -0.269 -0.045 -0.276 -0.246 -0.060 **0.263** 1.000
I0008 I0008 -0.179 -0.058 -0.091 -0.157 -0.171 0.090 **0.217** 1.000
I0009 I0009 -0.172 -0.141 -0.241 -0.194 -0.030 -0.197 0.063 **0.200** 1.000
I0010 I0010 -0.173 -0.162 -0.284 -0.265 0.016 -0.009 0.003 -0.102 -0.037 1.000
I0011 I0011 -0.253 -0.206 -0.257 -0.266 0.004 -0.122 -0.153 -0.085 -0.047 0.070 1.000
I0012 I0012 -0.227 -0.153 -0.271 -0.154 -0.065 -0.122 0.091 -0.126 0.026 0.000 **0.326** 1.000
I0013 I0013 0.083 -0.051 -0.015 -0.065 -0.165 -0.225 -0.159 -0.059 -0.065 -0.061 0.037 0.041 1.000
I0014 I0014 -0.161 -0.057 -0.167 -0.219 -0.073 -0.120 -0.156 -0.220 -0.114 0.021 0.003 -0.104 0.027 1.000
I0015 I0015 -0.112 -0.187 0.073 -0.043 -0.023 -0.174 -0.281 -0.156 -0.028 -0.118 -0.112 -0.224 0.024 **0.309** 1.000

**Table 2**. Raw score to the metric CIJSS

| Score | CIJSS_Metric |
| --- | --- |
| 0 | 0.0 |
| 1 | 4.1 |
| 2 | 6.7 |
| 3 | 8.3 |
| 4 | 9.5 |
| 5 | 10.5 |
| 6 | 11.4 |
| 7 | 12.1 |
| 8 | 12.8 |
| 9 | 13.5 |
| 10 | 14.1 |
| 11 | 14.6 |
| 12 | 15.1 |
| 13 | 15.6 |
| 14 | 16.1 |
| 15 | 16.6 |
| 16 | 17.0 |
| 17 | 17.5 |
| 18 | 17.9 |
| 19 | 18.4 |
| 20 | 18.8 |
| 21 | 19.3 |
| 22 | 19.8 |
| 23 | 20.3 |
| 24 | 20.7 |
| 25 | 21.3 |
| 26 | 21.8 |
| 27 | 22.3 |
| 28 | 22.9 |
| 29 | 23.4 |
| 30 | 24.0 |
| 31 | 24.6 |
| 32 | 25.2 |
| 33 | 25.9 |
| 34 | 26.5 |
| 35 | 27.2 |
| 36 | 27.8 |
| 37 | 28.5 |
| 38 | 29.2 |
| 39 | 30.0 |
| 40 | 30.7 |
| 41 | 31.4 |
| 42 | 32.2 |
| 43 | 33.0 |
| 44 | 33.7 |
| 45 | 34.5 |
| 46 | 35.3 |
| 47 | 36.2 |
| 48 | 37.0 |
| 49 | 37.9 |
| 50 | 38.9 |
| 51 | 39.9 |
| 52 | 40.9 |
| 53 | 42.1 |
| 54 | 43.4 |
| 55 | 44.8 |
| 56 | 46.4 |
| 57 | 48.3 |
| 58 | 50.8 |
| 59 | 54.4 |
| 60 | 60.0 |

**References**

1. Rasch G. Probabilistic models for some intelligence and attainment tests: ERIC; 1993.

2. Masters GN. A Rasch model for partial credit scoring. Psychometrika. 1982;47(2):149-74.

3. Tennant A, Küçükdeveci AA. Application of the Rasch measurement model in rehabilitation research and practice: early developments, current practice, and future challenges. Frontiers in Rehabilitation Sciences. 2023;4:1208670.

4. Teresi JA, Kleinman M, Ocepek‐Welikson K. Modern psychometric methods for detection of differential item functioning: application to cognitive assessment measures. Statistics in medicine. 2000;19(11‐12):1651-83.

5. Christensen KB, Makransky G, Horton M. Critical values for Yen’s Q 3: Identification of local dependence in the Rasch model using residual correlations. Applied psychological measurement. 2017;41(3):178-94.

6. Tesio L, Caronni A, Simone A, Kumbhare D, Scarano S. Interpreting results from Rasch analysis 2. Advanced model applications and the data-model fit assessment. Disability and rehabilitation. 2024;46(3):604-17.

7. Müller M. Item fit statistics for Rasch analysis: can we trust them? Journal of Statistical Distributions and Applications. 2020;7:1-12.

8. Smith Jr EV. Detecting and evaluating the impact of multidimensionality using item fit statistics and principal component analysis of residuals. Journal of applied measurement. 2002;3(2):205-31.

9. Gignac MA, Sutton D, Badley EM. Arthritis symptoms, the work environment, and the future: measuring perceived job strain among employed persons with arthritis. Arthritis Care & Research: Official Journal of the American College of Rheumatology. 2007;57(5):738-47.

10. Hammond A, Tennant A, Ching A, Parker J, Prior Y, Gignac MA, et al. Psychometric testing of the British‐English long‐term conditions job strain scale, long‐term conditions work spillover scale and work‐health‐personal life perceptions Scale in four rheumatic and musculoskeletal conditions. Musculoskeletal Care. 2023;21(4):1020-35.
